# Ablation margin quantification after thermal ablation of malignant liver tumors: How to optimize the procedure? A systematic review of the available evidence

**DOI:** 10.1016/j.ejro.2023.100501

**Published:** 2023-06-27

**Authors:** Pim Hendriks, Fleur Boel, Timo TM Oosterveer, Alexander Broersen, Lioe-Fee de Geus-Oei, Jouke Dijkstra, Mark C Burgmans

**Affiliations:** a Department of Radiology, Leiden University Medical Center, Leiden, the Netherlands; b LKEB Laboratory of Clinical and Experimental Imaging, Department of Radiology, Leiden University Medical Center, Leiden, the Netherlands; c Biomedical Photonic Imaging Group, University of Twente, the Netherlands

**Keywords:** Thermal ablation, RFA, MWA, Ablation margin quantification, Image co-registration, Tissue shrinkage

## Abstract

**Introduction:**

To minimize the risk of local tumor progression after thermal ablation of liver malignancies, complete tumor ablation with sufficient ablation margins is a prerequisite. This has resulted in ablation margin quantification to become a rapidly evolving field. The aim of this systematic review is to give an overview of the available literature with respect to clinical studies and technical aspects potentially influencing the interpretation and evaluation of ablation margins.

**Methods:**

The Medline database was reviewed for studies on radiofrequency and microwave ablation of liver cancer, ablation margins, image processing and tissue shrinkage. Studies included in this systematic review were analyzed for qualitative and quantitative assessment methods of ablation margins, segmentation and co-registration methods, and the potential influence of tissue shrinkage occurring during thermal ablation.

**Results:**

75 articles were included of which 58 were clinical studies. In most clinical studies the aimed minimal ablation margin (MAM) was ≥ 5 mm. In 10/31 studies, MAM quantification was performed in 3D rather than in three orthogonal image planes. Segmentations were performed either semi-automatically or manually. Rigid and non-rigid co-registration algorithms were used about as often. Tissue shrinkage rates ranged from 7% to 74%.

**Conclusions:**

There is a high variability in ablation margin quantification methods. Prospectively obtained data and a validated robust workflow are needed to better understand the clinical value. Interpretation of quantified ablation margins may be influenced by tissue shrinkage, as this may cause underestimation.

## Highlights

• There is a high variability in ablation margin quantification methodology.
• A minimal ablation margin of ≥ 5 mm is aimed for in most studies.
• Differences in image co-registration and segmentation algorithms were found.
• Tissue shrinkage may cause underestimation of obtained ablation margins.

## 1 Introduction

Thermal ablation is an effective treatment for primary and secondary liver tumors [1], [2], [3]. For tumors of limited size (≤2 cm) thermal ablation using radiofrequency ablation (RFA) or microwave ablation (MWA) is a first line therapy, particularly in patients with co-morbidity, underlying liver cirrhosis and/or centrally located tumors. Nevertheless, surgical resection is generally considered to be more effective, as thermal ablation is associated with higher local tumor progression (LTP) rates. To minimize the risk of LTP after thermal ablation, complete tumor ablation with sufficient ablation margins is essential. The correlation between ablation margins and LTP was first demonstrated in 2008 by Kei et al. [4]. Later, this was confirmed by other large trials [5], [6], [7].

Most commonly, ablation margins after thermal ablation are assessed by side-by-side comparison of pre- and post-ablation cross-sectional images. This method is usually based on visual assessment, i.e. eye-balling, but may be aided by two-dimensional measurements using anatomical landmarks on both scans. The use of software-assisted quantitative assessment of ablation margins has gained interest in literature over the last years [6], [7], [8], [9]. Several studies indicate it could contribute to better determine technical success of thermal ablation treatments and estimate the risk of LTP [7], [8], [9]. However, there is wide variation in methods used for margin quantification and the optimal method has not yet been established.

Ablation margin quantification is performed using software with specific segmentation and image co-registration algorithms. The co-registration algorithms may differ by design as co-registration can be performed either in a rigid or non-rigid way. In rigid co-registration, the images are registered using only rotation and translation of the images whereas non-rigid co-registration also allows deformation of the images. Besides the differences between rigid and non-rigid co-registration, the co-registration could be performed manually, semi-automatically or fully automatically. Other differences may be with respect to volume of interest selection or usage of landmarks.

Besides the more technical variety among co-registration algorithms, patient and treatment related factors may affect the result of ablation margin quantification. Differences in respiration mode and patient positioning may cause considerable variation in the shape and position of the liver between the pre- and post-ablation scans. Moreover, tissue shrinkage as a direct result of tissue heating possesses an important challenge on ablation margin interpretation [10]. As the ablated tissue tends to shrink during thermal ablation, the ablation margins may be underestimated. Unfortunately, the degree and direction of tissue shrinkage is unpredictable [10].

Quantitative ablation margin assessment holds great promise as a tool to better predict patients at risk for LTP after thermal ablation. The aim of this systematic review is to create an overview of the current evidence with respect to qualitative and quantitative evaluation methods of ablation margins, image processing tools, and the potential influence of tissue shrinkage occurring during thermal ablation.

## 2 Methods

### 2.1 Search strategy

The electronic database Medline was searched on 01/02/2021 for all studies describing “image segmentation”, “image registration”, “ablation margins”, “treatment success” or “tissue shrinkage” during treatment of liver tumors using thermal ablation techniques, i.e. “RFA” or “MWA”, since 01/01/2009 as techniques have constantly been improving and the quality of ablation of > 12 years old was not considered representative. The full search term used can be found in Appendix A. Articles were sequentially evaluated based on title, abstract and full text for meeting all in- and exclusion criteria. The literature search, study selection, data extraction and study quality assessment were independently conducted by two reviewers (P.H. and F.B.). Any disagreements were resolved in consensus.

### 2.2 Exclusion criteria

Articles were excluded if they did not relate to percutaneous thermal ablation of malignant liver tumors with RFA or MWA; if surgical resection was performed; and if the aim of the article was to evaluate combination therapy with ablation and trans-arterial or systemic therapy. Articles related to liver segmentation or co-registration were excluded if they did not define the segmentation or co-registration method used; and if ultrasound (US), positron emission tomography (PET), or single photon emission computed tomography (SPECT) images were used for image segmentation or co-registration. Articles using hybrid imaging modalities were not excluded if tumor and/or ablation zone segmentation was performed using (contrast-enhanced) CT or MRI. Articles related to evaluation of ablation margins were excluded if they did not provide a definition for technical success or minimal ablation margins. Finally, systematic reviews, reviews, letters to the editor, conference abstracts and full-text articles in other languages than English were excluded. References of systematic reviews and reviews were evaluated for further inclusion of articles missed in the initial search.

### 2.3 Data extraction

For each article, the following information was extracted if present: first author, publication year, journal, study type, imaging modality, tumor type, mean tumor size, number of subjects and tumors, ablation method, software used, intended minimal ablation margin (MAM), LTP rate, method of MAM determination, segmentation method, co-registration method, other treatment success outcome measures, and validation of segmentation and registration.

## 3 Results

**Fig. 1:**
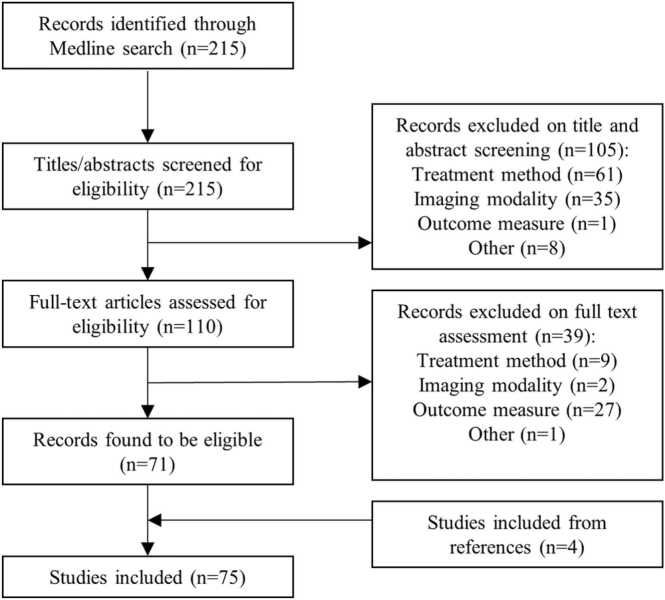
Overview of the article selection process, specified per step.

The search strategy initially resulted in 215 articles, that were screened by title and abstract (Fig. 1). Subsequently, 110 articles were analyzed in full-text for eligibility, resulting in the inclusion of 71 articles. Another 4 articles were included from references of (systematic) reviews. Eventually, a total of 75 articles were included in this review, see Fig. 1. Thirty-one articles described a method for determination of technical success or measurement of MAM [5], [6], [7], [8], [9], [11], [12], [13], [14], [15], [16], [17], [18], [19], [20], [21], [22], [23], [24], [25], [26], [27], [28], [29], [30], [31], [32], [33], [34], [35], [36]. Thirteen articles described segmentation methods for segmentation of the tumor and the ablation zone [8], [9], [22], [31], [37], [38], [39], [40], [41], [42], [43], [44], [45]. Twenty-five articles reported on co-registration methods for either pre- and postinterventional image co-registration, or pre- and intraoperative image co-registration [6], [7], [8], [9], [12], [24], [26], [27], [28], [31], [32], [34], [36], [42], [44], [45], [46], [47], [48], [49], [50], [51], [52], [53], [54]. Finally, ten articles evaluated tissue shrinkage due to thermal ablation [10], [55], [56], [57], [58], [59], [60], [61], [62], [63].

### 3.1 Clinical studies

In total, 58 clinical studies with 4311 tumors were included in the results. RFA was the ablation method used most frequently and HCC patients (n = 3431 tumors) formed the main population in most studies. Intrahepatic cholangiocarcinoma (n = 57) and hepatic metastases from other primary origin (predominantly colorectal cancer, n = 456) were other pathologies included. All studies were performed in a single center and most of them had a retrospective study design. A high variety in population size was found (7–211, median: 36.5). Mean or median lesion sizes were < 30 mm for all clinical studies. An overview of all included clinical studies can be found in Table 1.

**Table 1:** Characteristics for all clinical studies included.

Authors	Study type (Retrospective R, Prospective P)	Ablation method	Tumor Type	Number of patients (tumors)	Tumor size	Intended MAM
Abdel-Rehim M et al.[11]	R	RFA and MWA	HCC (17), CRLM (3), BCLM (1), CCA (2)	23 (23)	Range 8–40 mm	≥ 5 mm
An C et al.[12]	R	MWA	HCC	141 (141)	Mean 23 mm ± 9 mm	≥ 5 mm
Beyer LP et al.[64]	R and P	MWA	HCC (20), CRCM (16)	36 (36)	Mean 21.2 mm	-
Biondetti P et al.[13]	R	MWA	HCC	74 (74)	Mean 17.1 mm, range 7 – 30 mm	≥ 5 mm
Boulkhrif H et al.[46]	R	RFA and MWA	HCC (35), CRLM (16), neuroendocrine (3), gastric (1), BCLM (1)	35 (56)	Mean 20.4 ± 9.4 mm, range 6.1–60 mm; median 18.3 mm	-
Cao F et al.[65]	R	MWA	MLM (melanoma liver metastases)	7 (22)	Median 16.37 mm, range 6.66 – 43.72 mm	-
Cha DI et al.[66]	R	RFA	HCC	146 (146)	Median 16 mm, range 7–42 mm	≥ 5 mm
Choi JW et al.[67]	P	RFA	HCC	79 (98)	Mean 19 ± 7 mm	≥ 5 mm
Choi JW et al.[14]	P	RFA	HCC	77 (86)	Mean 16.5 mm	≥ 5 mm
El-Gendi A et al.[68]	P	RFA	HCC	24 (24)	Mean 20.4 ± 4.4 mm	≥ 10 mm
Fukuda K et al.[15]	P	RFA	HCC	76 (85)	Median 15 mm, range 8 − 30 mm	≥ 10 mm
Fumarola EM et al.[69]	R	MWA	HCC	50 (50)	Mean 17.6 mm, range 7 – 35 mm	-
Hame Y et al.[39]	R	RFA	-	9 (11)	< 5 mm	-
Hendriks P et al.[8]	R	RFA	HCC	25 (25)	Median 20 mm, range 12 – 45 mm	≥ 5 mm
Hocquelet A et al.[22]	R	RFA	HCC	16 (16)	Mean 29 mm	≥ 5 mm
Iwazawa J et al.[16]	R	RFA	HCC (8), metastatic (4)	12 (12)	Mean 16.3 mm, range 8 – 20 mm	≥ 5 mm
Iyer RS et al.[44]	R	RFA	HCC (20), metastatic (19)	29 (39)	-	≥ 10 mm
Jiang C et al.[23]	R	RFA	HCC	134 (159)	Mean 20 ± 9 mm, range 10 – 49 mm	≥ 5 mm
Kamei S et al.[70]	R	RFA	HCC	19 (22)	Mean 17.5 ± 7.9 mm, range 9 – 34 mm	≥ 5 mm
Kang TW et al.[71]	R	RFA	HCC	211 (211)	Mean 21 mm	≥ 5 mm
Kaye EA at al.[45]	R	RFA	CRLM	72 (93)	Mean 18 mm, range 6–55 mm	-
Keil S et al.[40]	R	RFA	BCLM (15), CRLM (35)	25 (50)	-	-
Keil S et al.[41]	R	RFA	BCLM (15), CRLM (35)	25 (50)	Mean 23 mm	-
Kim KW et al.[49]	R	RFA	HCC	31 (38)	Mean 19 mm, range 10 – 35 mm	-
Kim SM et al.[17]	P	RFA	HCC	33 (42)	Mean 15.8 ± 5.9, range 7–33 mm	-
Kim YS et al.[6]	P	RFA	HCC	103 (110)	Mean 27 ± 6 mm, range 21 – 48 mm	≥ 5 mm
Kobe A et al.[24]	R	RFA	HCC	39 (43)	Median 16.9 mm, range 14.6 – 22.4 mm	-
Koh YH et al.[18]	R	RFA	HCC	64 (75)	Mean 14.0 ± 4.6 mm, range 10–37 mm	≥ 5 mm
Laimer G et al.[7]	R	RFA	HCC	110 (176)	Mean 25.2 ± 14.9 mm	-
Lee JK et al.[63]	R	RFA and MWA	HCC (49) and metastatic (26)	65 (75)	Range 10 – 65 mm	-
Lee MW et al.[72]	R	RFA	HCC	18 (19)	Mean 25 mm, median 23 mm, range 20 – 42 mm	≥ 5 mm
Li X et al.[73]	R	MWA	NPC metastases	18 (24)	Maximum diameter of 42 mm	≥ 5 mm
Liao M et al.[25]	P	RFA	HCC	80 (83)	Mean 24.5 mm	≥ 5 mm
Liu ZY et al.[74]	P	RFA	CRLM	12 (20)	Mean 28 mm, range 15 – 52 mm	≥ 5 mm
Makino Y et al.[27]	R	RFA	HCC	85 (94)	Mean 14.0 ± 5.2 mm	-
Makino Y et al.[26]	R	RFA	HCC	67 (92)	Median 12.9 mm, range 4.8 – 41.4 mm	-
Motoyama T et al.[19]	R	RFA	HCC	66 (95)	Median 18 mm, range 7 – 33 mm	≥ 5 mm
Park J et al.[28]	R	RFA	HCC	178 (178)	Mean 17.3 ± 6.1 mm	-
Park SI et al.[75]	R	RFA	HCC (15), rectosigmoid metastases (1), CCA (1)	15 (17)	Mean 15.68 ± 5.29 mm, range 10 – 26 mm	≥ 10 mm
Park Y et al.[20]	R	RFA	HCC	146 (167)	Mean 19 mm, median 18 mm, range 8 – 40 mm	≥ 5 mm
Passera K et al.[42]	R	RFA	HCC (5), metastatic (5)	10 (10)	Range 10–40 mm	-
Ringe KI et al.[21]	R	RFA and MWA		32 (48)	Mean 24 mm, range 9 – 64 mm	-
Sakakibara M et al.[29]	R	RFA	HCC	84 (139)	Mean 13.8 ± 4.6 mm	≥ 5 mm
Shin S et al.[30]	P	RFA	HCC	150 (150)	Mean 19.5 ± 7.9 mm	≥ 5 mm
Sibinga Mulder BG et al.[9]	R	RFA	CRLM	29 (29)	Median 22 mm, range 8 – 22 mm	≥ 5 mm
Solbiati M et al.[31]	R	MWA	HCC	50 (90)	Mean 27 ± 20 mm	≥ 5 mm
Sotirchos VS et al.[35]	P	RFA	CRLM	47 (67)	Mean 21 mm, range 6 – 43 mm	≥ 5 mm
Takeyama N et al.[32]	R	RFA	HCC	29 (59)	Mean 11.2 ± 4.4 mm, range 5 – 24 mm	≥ 3 mm
Van Tilborg AA et al.[76]	R	RFA and MWA	HCC (7), CRLM (29), CCA (2)	20 (38)	Mean 22 mm	≥ 5 mm
Tinguely P et al.[33]	R	MWA	HCC (174), CRLM (87), NET (17), other (23)	153 (301)	Median 15 mm, IQR 11–21 mm	≥ 5 mm
Vandenbroucke F et al.[34]	R	RFA	CRLM (16), melanoma metastases (3), BCLM (1)	20 (45)	Mean 18.6 mm, median 18 mm, range 6 – 41 mm	-
Vo Chieu VD et al.[77]	R	MWA	HCC/CCA* (97), metastases (77)	94 (174)	Median 19 mm, range 4 – 51 mm	≥ 5 mm
Vo Chieu VD et al.[43]	R	MWA	HCC (17), CCA (3), metastases (20)	27 (40)	Mean 17.3 ± 6.5 mm, range 6 – 31.5 mm	≥ 5 mm
Wang XL et al.[52]	R	RFA	HCC	52 (62)	Mean 20 ± 10 mm, range 10 – 31 mm	-
Yan Y et al.[78]	R	RFA and MWA	Primary (7), secondary (7)	12 (14)	Mean 16.6 ± 13.4 mm, range 3 – 45 mm	≥ 5 mm
Yoon JH et al.[79]	P	RFA	HCC	36 (43)	Mean 24.5 mm, range 20 – 47 mm	≥ 5 mm
Yoon JH et al.[36]	P	RFA	HCC	68 (88)	Mean 16 mm ± 6 mm, range 6 – 32 mm	≥ 5 mm
Zhang Q et al.[80]	R	RFA	HCC (29), CCA (1), CRLM (9), OCLM (2), PNET metastases (1)	37 (37)	Mean 26.6 ± 15.1 mm, range 9.1–66.7 mm	≥ 5 mm

RFA: radiofrequency ablation. MWA: microwave ablation. HCC: hepatocellular carcinoma. CRLM: colorectal liver metastasis. BCLM: breast cancer liver metastasis. CCA: cholangiocarcinoma. GEP-NET: gastroenteropancreatic neuroendocrine tumors. NET: neuroendocrine tumors. NPC: nasopharyngeal carcinoma. OCLM: ovarian cancer liver metastases. PNET: primitive neuroectodermal tumor. MAM: minimal ablative margin. *Not further specified, considered 50% HCC and 50% CCA

### 3.2 MAM assessment

In general, three ways of pre- and post-ablation imaging assessment were identified from the results, see Table 2. Analysis with side-by-side projection of pre- and post-scans was performed in 14/31 studies [5], [8], [13], [14], [15], [16], [17], [18], [19], [20], [21], [27], [28], [35]. As part of this assessment MAM was determined in the axial plane (n = 13) [5], [8], [13], [15], [16], [17], [18], [19], [20], [21], [26], [28], [35] or in the coronal and sagittal imaging planes too (n = 10) [5], [8], [13], [16], [17], [19], [20], [26], [28], [35]. Manual 2D measurements using anatomical landmarks were performed to quantify the ablation margins in these studies using mostly anatomical landmarks. In 21/31 studies co-registration software for MAM quantification was used [6], [7], [8], [9], [11], [12], [22], [23], [24], [25], [26], [27], [28], [29], [30], [31], [32], [33], [34], [35], [36]. The software used can further be categorized into a) non-dedicated co-registration software combined with manual measurements (n = 17) [6], [7], [8], [9], [11], [12], [22], [24], [25], [26], [27], [28], [29], [30], [32], [33], [34], [36] and b) dedicated MAM quantification software that allows segmentation of tumor and ablation necrosis (n = 3) [22], [23], [31].

**Table 2:** Methodology of ablation margin analysis.

Article	Modality	Pre- and post-ablation image analysis			MAM measurements			Other treatment success measures	
		Side-to-side	Co-registration software	Ablation margin quantification software	In axial plane	In 3 orthogonal planes	In 3D (oblique angles)	Ablation surface area	Volumetric ablation margin data
Biondetti P et al.[13]	CT and MRI	+	-	-	+	+	-	-	-
Choi JW et al.[14]	CT and MRI	+	-	-	-	-	-	-	-
Fukuda K et al.[15]	CT	+	-	-	+	-	-	-	-
Iwazawa J et al.[16]	CT	+	-	-	+	+	-	-	-
Kim SM et al.[17]	CT and MRI	+	-	-	+	+	-	-	-
Koh YH et al.[18]	CT	+	-	-	+	-	-	-	-
Motoyama T et al.[19]	CT	+	-	-	+	+	-	+	-
Park Y et al.[20]	CT	+	-	-	+	+	-	+	-
Ringe KI et al.[21]	CT and MRI	+	-	-	+	-	-	-	-
Wang X et al.[5]	CT	+	-	-	+	+	-	-	-
Sotirchos et al.[35]	CT	+	-	-	+	+	-	-	-
Abdel-Rehim M et al.[11]	CT and CBCT	-	+	-	+	+	-	-	-
An C et al.[12]	MRI	-	+	-	+	+	+	-	+
Hendriks P et al.[8]	CT	+	+	-	+	+	+	-	-
Kim YS et al.[6]	CT	-	+	-	+	+	-	-	-
Kobe A et al.[24]	CT and MRI	-	+	-	-	-	+	-	+
Laimer G et al.[7]	CT	-	+	-	-	-	+	-	-
Liao M et al.[25]	CT	-	+	-	+	+	-	-	-
Makino Y et al.[27]	CT	-	+	-	+	-	-	-	-
Makino Y et al.[26]	CT and MRI	+	+	-	+	+	-	-	-
Park J et al.[28]	CT and MRI	+	+	-	+	+	+	-	-
Sakakibara M et al.[29]	CT and MRI	-	+	-	+	+	-	-	-
Shin S et al.[30]	CT	-	+	-	+	+	-	-	-
Takeyama et al.[32]	MRI	-	+	-	-	-	-	-	-
Tinguely P et al.[33]	CT	-	+	-	-	+	-	-	-
Vandenbroucke F et al.[34]	CT	-	+	-	+	+	-	-	-
Hocquelet A et al.[22]	MRI	-	+	+	-	-	+	+	+
Jiang C et al.[23]	CT	-	+	+	-	-	+	-	-
Sibinga Mulder BG et al.[9]	CT	-	+	-	-	-	+	-	-
Solbiati M et al.[31]	CT	-	+	+	-	-	+	-	+
Yoon JH et al.[36]	CT and MRI	-	+	-	-	-	+	-	-

CT: computed tomography. CBCT: cone-beam computed tomography. MRI: magnetic resonance imaging. MAM: minimal ablation margin.

Euclidian distance measurements were used to quantify the MAM in 3D in case of dedicated MAM quantification software. In non-dedicated co-registration software, either a visual assessment (n = 1) [30], in-plane measurements (n = 10) [6], [11], [25], [26], [27], [28], [29], [30], [33], [34] or 3D MAM measurements (n = 6) [7], [8], [9], [12], [24], [36] were used.

**Fig. 2:**
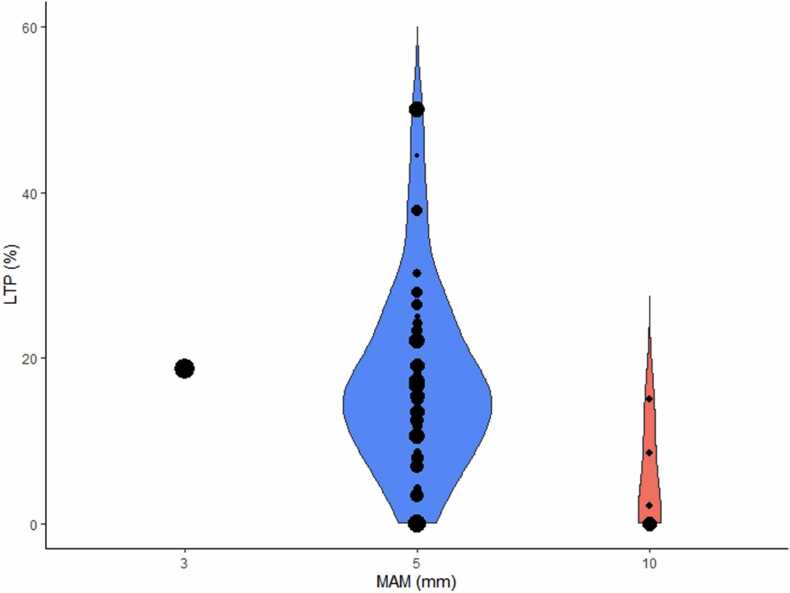
Violin plot showing the local tumor progression (LTP) rates for different intended minimal ablation margins (MAM). Horizontal width of the plot represents the density of the data along the Y-axis. Each individual dataset is represented as a dot, where larger dots represent studies with more tumors treated.

In all studies, the MAM was expressed as the smallest distance from the tumor boundary to the nearest border of the ablation zone. In general, the intended MAM was ≥ 5 mm, as can be seen in Table 1. In a few studies additional quantification measures were used, such as the coverage of the tumor by the ablation zone, or the extent that a 5 mm ablation margin was reached in all directions. In 36 studies, the quantified MAM was correlated with the occurrence of LTP. Fig. 2 shows the correlation between the intended MAM and the occurrence of LTP. In one study immunohistology of a post-ablation biopsy was correlated with the occurrence of LTP [35].

### 3.3 Segmentation methods

Table 3 describes the different methods used for segmentation of the tumor and ablation zone. Semi-automatic segmentation methods were used in 12/13 studies [8], [9], [22], [31], [37], [38], [39], [40], [41], [42], [43], [44] and manual segmentation was used in only one study [45]. Semi-automatic segmentation methods used included edge detection [8], [9], [44], region growing based algorithms [22], [40], [41], [45], and machine learning based algorithms involving classification [39] and clustering [37], [38], [42]. In four of these papers in-house segmentation software was used [37], [38], [39], [42] and in the other studies commercially available software [8], [9], [22], [31], [40], [41], [43], [44].

**Table 3:** Specifications of segmentation algorithms used.

Used in article	Used imaging modality	Commercial software	Used for: T = tumor	Automation of segmentation	(Semi-) automatic segmentation algorithm used	Evaluation of segmentation	Performance
			AZ = ablation zone B = both	M = manual SA = semi-automatic FA = fully automatic			
Egger J et al.[38]	CT	-	AZ	SA	Interactive, graph-based contouring approach with preference for spherically shaped regions.	Dice similarity coefficient (DSC)	DSC = 77.1
Egger J et al.[37]	CT	-	AZ	SA	Interactive, graph-based contouring approach with preference for spherically shaped regions.	Dice similarity coefficient (DSC)	DSC = 77.0
Hame Y et al.[39]	CT	-	T	SA	Non-parametric intensity distribution estimation and hidden Markov measure field model, with application of a spherical shape prior.	Volumetric overlap error (VOE), Volume difference (VD), Symmetric surface distance (SD), Root mean square symmetric volume distance (RD), Maximum symmetric surface distance (MD)	OE= 29.60 ± 5.61 VD= 17.75 ± 11.40 SD= 0.89 ± 0.31 RD= 1.24 ± 0.42 MD= 5.12 ± 2.75
Hendriks P et al.[8]	CT	Mirada RTx	B	SA	Greyscale based delineation tool	Cohen’s kappa statistics	K= 0.88
Hocquelet A et al.[22]	MRI	ITK-SNAP freeware	B	SA	Region-competition snakes	-	-
Iyer RS et al.[44]	CT and MRI	Adope Photoshop CS v8.0	B	M	Magnetic lasso tool	-	-
Kaye EA et al.[45]	CT and MRI	MIM MEASTRO	B	SA	Seed point based region growing	-	-
Keil S et al.[41]	CT	SyngoCT Oncology, Siemens Healthcare	B	SA	Supervised seeded region growing algorithm	Lin’s concordance correlation coefficient for volume (V), CT value (D)	V_tumor_: 0.98 (0.97 – 0.99) D_tumor_: 0.90 (0.83 – 0.94) V_ablation_: 0.99 (0.95 – 0.99) D_ablation_: 0.76 (0.62 – 0.85)
Keil S et al.[40]	CT	Syngo Oncology NNWP VE 31 H (Siemens Medical Solutions)	B	SA	Supervised seeded region growing algorithm	Lin’s concordance correlation coefficient for volume (V), CT value (D)	V_tumor_: 0.98 (0.97 – 0.99) D_tumor_: 0.90 (0.83 – 0.94) V_ablation_: 0.99 (0.98 – 0.99) D_ablation_: 0.76 (0.62 – 0.85)
Passera K et al.[42]	CT	-	B	SA	Clustering based algorithm	Percentage Match (PM), Positive predictivity (PP), Negative predictivity (NP), Specificity (SPEC),	PM_tumor_: 92% PP_tumor_: 95% NP_tumor_: 94% SPEC_tumor_: 96% PM_ablation_: 90% PP_ablation_: 96% NP_ablation_: 93% SPEC_ablation_: 97%
Sibinga Mulder BG et al.[9]	CT	Mirada RTx	B	SA	Greyscale based delineation tool	-	-
Vo Chieu VD et al.[43]	CT	SAFIR (Software Assistant For Interventional Radiology)	AZ	SA	Combined threshold-based and model-based morphological processing.	Pearson correlation	r_max_diameter1_: 0.715 r_max_diameter2_: 0.659 r_ellipticity_: 0.707 r_volume:_ 0.978
Solbiati M et al.[31]	CT	Ablation-fit	B	SA		-	-

CT = computed tomography, MRI = magnetic resonance imaging

The accuracy of segmentation was qualitatively assessed by radiologists in all studies. Quantitative inter- or intra-observer agreement methods were used in eight studies, comparing semi-automatic segmentation with manual segmentation of an observer or the interobserver agreement between manual segmentation of multiple observers. Outcome measures used were the dice similarity coefficient (DSC) [37], [38], volumetric overlap error [39], volume difference [39], average symmetric surface distance [39], root mean square symmetric surface distance [39], maximum symmetric surface distance [39], Lin’s concordance correlation coefficient [40], [41], percentage match [42], positive and negative predictivity [42], specificity [42], and Pearson correlation [43].

### 3.4 Registration and MAM quantification software

Table 4 provides an overview of the different software used for co-registration and MAM quantification. CECT-images were used for co-registration in 22/25 studies [6], [7], [8], [9], [24], [26], [27], [28], [31], [34], [36], [42], [44], [45], [46], [48], [50], [51], [53], [54], and MRI-images were used in 10/25 studies [12], [24], [26], [28], [32], [36], [44], [45], [52], [53]. In-house developed software was used in 9/25 studies [12], [42], [46], [47], [48], [50], [51], [53], [54].

**Table 4:** Specifications of software used for co-registration and ablation margin quantification.

Name of software	Used in article	Used imaging modality	Commercially available	Dedicated for ablation margin quantification	Co-Registration method	Automation of co-registration	Uses landmarks	Evaluation of co-registration
					R = rigid NR = non-rigid	M = manual SA = semi-automatic FA = fully automatic	+ = landmarks based o = optional - = no landmarks used	
HepaCare	Kobe A et al.[24]	CT/MRI	Prototype	-	NR	(S)A	O	Visual
HepaCare	Kim KW et al.[49]	CT-CT	Prototype	-	NR	(S)A	O	Visual; co-registration error
HepaCare	Yoon JH et al.[36]	CT-MRI	Prototype	-	NR	(S)A	O	Visual
HepaCare 3.0	Park J et al.[28]	CT-MRI	Prototype	-	NR	-	-	-
In house	An C et al.[12]	MRI-MRI	-	-	NR	FA	-	Similarity metric between paired regions
In house	Boulkhrif H et al.[46]	CT-CT	-	-	NR	SA	-	Target co-registration error
In house	Gunay G et al.[48]	CT-CT	-	-	NR	(S)A	O	DICE overlap; Average surface distance
In house	Passera K et al.[42]	CT-CT	-	-	NR	FA	-	-
In house	Wei D.[53]	CT-MRI/CT-CT	-	-	R and NR	FA	-	Dice ratios and target co-registration error
In house	Gunay G et al.[47]	CT-CT	-	-	NR	SA	+	Average surface distance
In house	Luu HM et al.[50]	CT-CT	-	-	NR	FA	-	Dice similarity coefficient (DSC), mean surface distance (MSD), mean corresponding difference (MCD) between landmarks.
In house	Luu HM et al.[51]	CT-CT	-	-	NR	SA	O	Dice similarity coefficient (DSC), mean surface distance (MSD), mean corresponding difference (MCD) between landmarks.
In house	Luu HM et al.[54]	CT-CT	-	-	NR	SA	-	Dice Similarity Coefficient
Integrated Registration, GE Healthcare	Makino Y et al.[27]	CT-CT	+	-	R	SA	-	Landmark based co-registration error
Integrated Registration, GE Healthcare	Makino Y et al.[26]	CT-CT/MRI-MRI	+	-	R	M	+	Landmark based co-registration error
Integrated Registration, GE Healthcare	Vandenbroucke F et al.[34]	CT-CT	+	-	R	(S)A	O	Visual
MIM MAESTRO	Kaye EA et al.[45]	CT-CT/CT-MRI	+	-	R	SA	O	Visual, co-registration error
Mirada RTx	Hendriks P et al.[8]	CT-CT	+	-	NR	SA	O	Visual
Mirada RTx	Sibinga Mulder BG et al.[9]	CT-CT	+	-	NR	SA	+	Visual
MyLab Twice, Esoate	Wang XL et al.[52]	MRI-MRI	+	-	R	M	+	Visual
Syngo.via VB20A, Siemens Healthineers	Laimer G et al.[7]	CT-CT	+	-	R	(S)A	O	Visual
Virtual Place Advanced Plus version 2.03 (CT workstation)	Kim YS et al.[6]	CT-CT	+	-	R	M	+	Visual
Volume Analyzer Synapse VINCENT, version 5.1, Fujifilm Medical Systems,	Takeyama N et al.[32]	MRI-MRI	+	-	R	(S)A	O	Visual
-	Iyer RS et al.[44]	CT-CT/MRI-MRI	-	-	R	M	+	Visual
Ablation-Fit	Solbiati M et al.[31]	CT-CT	+	+	R and NR	A	-	-

CT= computed tomography, MRI = magnetic resonance imaging,

Rigid co-registration algorithms that only allowed for translation and rotation of images for optimal co-registration were used in 11/25 studies [6], [7], [26], [27], [31], [32], [34], [44], [45], [52], [53]. Reasons for choosing rigid co-registration could be speed, and availability. In 14/25 studies, non-rigid co-registration algorithms were used that also allowed for deformation of the scans to locally optimize the co-registration [8], [9], [12], [24], [28], [31], [36], [42], [46], [47], [48], [49], [50], [51], [53], [54]. Reasoning behind the choice of a non-rigid approach were to allow for local liver deformations and to reduce the influence of respiratory motion, adjacent organ movement, heart pulsations and patient positioning. In two studies, both co-registration methods were used [31], [53].

Anatomical landmark placement on the liver surface, hepatic arteries, portal veins and hepatic veins near the tumor were (optionally) used as input parameters in 16/25 different studies [6], [7], [8], [9], [24], [26], [32], [34], [36], [44], [45], [47], [48], [49], [51], [52]. Placement of anatomical landmarks near the tumor and ablation necrosis was used for local optimization of the image co-registration.

Fully automated co-registration algorithms were used in 6/25 studies [12], [26], [31], [42], [50], [53]. Semi-automatic co-registrations algorithms were used in 14/25 studies [7], [8], [9], [24], [32], [34], [36], [45], [46], [47], [48], [49], [51], [54]. Manual translation and rotation was possible to adjust the co-registrations in these software packages. Three commercial software packages were used that only allowed for manual co-registration [6], [26], [52]. One software platform was commercially available and dedicated to ablation margin quantification [31].

Quality of co-registration was described in 22/25 studies [6], [7], [8], [9], [12], [24], [26], [27], [32], [34], [36], [44], [46], [47], [48], [49], [50], [51], [52], [53], [54]. This was qualitatively scored in 12/22 software packages using e.g. a three- or five-points scale [6], [7], [8], [9], [24], [32], [34], [36], [44], [45], [49], [52]. Quantitative quality assessment measures included distances between one or multiple pairs of landmarks, and distances between surface areas. The Dice similarity coefficient between segmented volumes was used as another quantitative quality assessment tool.

### 3.5 Tissue shrinkage

Tissue shrinkage was evaluated using ex vivo bovine or porcine livers [10], [55], [56], [57], [58], [59], [60], in vivo porcine livers [61], [62] or pre- and post-ablation imaging of patients with HCC or metastases [63]. In the ex vivo animal models, the liver was divided in test samples, after which ablation was performed using either RFA or MWA. In the in vivo animal model, the ablation was performed in different segments of the liver. The samples consisted of normal liver parenchyma without tumors. Ablation times ranged from 1 min to 20 min, with power settings between 20 and 200 W. Tissue shrinkage was measured through the dimensions of the samples pre- and post-ablation, or the displacement of markers inserted into the tissue sample. Tissue shrinkage was expressed as the contraction ratio, or contraction measured in percentage, see Table 5. Noteworthy, in the study by Weiss et al. the contraction was expressed as planar strain, which showed tissue dilatiation for ablation times < 10 min [59].

**Table 5:** Tissue shrinkage.

Authors	Ablation method	Study subjects (number of tests)	Contraction ratio	Tissue shrinkage [%]
Amabile C et al.[55]	RFA	Ex vivo bovine (n = 6)	0.88 ± 0.05	
	MWA	Ex vivo bovine (n = 4)	Radial: 0.83 Longitudinal: 0.82	Radial: 20.5 Longitudinal: 22.5
Brace CL et al.[56]	RFA	Ex-vivo porcine (n = 20)		In 1 diameter: 15
	MWA	Ex-vivo porcine (n = 8)		In 1 diameter: 30
Bressem KK et al.[61]	MWA	In-vivo porcine (n = 10)		4
Erxleben C et al.[62]	MWA	In-vivo porcine (n = 19)		1–12
Farina L et al.[10]	MWA	Ex-vivo bovine Ex-vivo turkey muscle (total: n = 119)		28–74
Lee JK et al.[63]	MWA	In-vivo human (n = 31)	Absolute ablation zone: 2.45 ± 0.47 Absolute tumor: 2.37 ± 0.28 mm	
	RFA	In-vivo human (n = 44)	Absolute ablation zone: 0.94 ± 0.38 mm Absolute tumor: 0.55 ± 0.26 mm	
Liu D et al.[57]	MWA	Ex-vivo bovine (n = 6)		Radial: 10 Longitudinal: 20 Volumetric:40
Liu D et al.[60]	MWA	Ex-vivo porcine (n = 16)		Radial: 11–35
Rossmann C et al.[58]	RFA	Ex-vivo porcine (n = 35)		12.3 – 21.7
Weiss N et al.[59]	MWA	Ex-vivo porcine (n = 16)	Planar strain: 10 min: 0.97 ± 0.02 1,2,3, 6 min: all > 1	

RFA = radiofrequency ablation, MWA = microwave ablation

Measurements in the in vivo human study were performed using landmarks on both pre- and post-ablation images, by two radiologists. A relative ablation zone contraction of 7.11% (+/- 13.3) and 2.39% (+/- 12.7), and tumor contraction of 9.95% (+/- 10.4) and 1.31% (+/- 13.2) were found for MWA and RFA, respectively [63].

## 4 Discussion

Ablation margin quantification has been a topic of high interest in literature. In this systematic review, we have evaluated clinical study methodology, MAM quantification software methods, imaging co-registration methods, segmentation methods and tissue shrinkage. In general, a high variety in methodology was found between different studies.

With respect to the clinical studies, a MAM of ≥ 5 mm was intended mostly, in accordance with ablation guidelines [81]. Although the studies were very heterogeneous, and only limited data were available of studies with an intended MAM of ≥ 3 mm and ≥ 10 mm, LTP rates tended to decrease at larger intended MAM.

In studies that aimed at retrospective quantification of the ablation margins, the properties of the ablation margin quantification tools or software were evaluated. The MAM (i.e. smallest distance between outer boundaries of tumor and ablation zone) was the outcome measure used in all studies. Only 3 studies used other additional outcome measures, such as ablation surface area or volumetric data. In a limited number of studies, the MAM could also be quantified in 3D rather than the standard orthogonal image planes. With the emerging field of dedicated ablation margin quantification software and incorporation of ablation margin quantification in clinical trials, it is expected that this more thorough analysis will become the new standard.

Segmentation of tumor and ablation zone plays a major role in objectively quantifying ablation margins. Several segmentation algorithms were used in the included studies, most of them were semi-automatic and based on underlying grey-scale or region-growing algorithms. Multiple methods were used to validate segmentations among different interpreters or against a golden standard. Although the results of these validations are not directly comparable, the overall performance seems good. To be better able to compare the robustness and accuracy of each segmentation tool, a standardized validation method would be needed, despite their specific advantages and disadvantages. The DSC is suitable for comparing two segmentations based on their overlap, but its sensitivity is dependent on the size of the segmented structure. Besides the technical aspects of segmentation, several clinical implications should be taken into consideration. The size and shape of a tumor may appear differently on arterial and venous phases. Choosing the right scan phase is therefore crucial for obtaining the correct ablation margin. Moreover, for a smooth incorporation in the clinical workflow it is important that segmentation algorithms are fast, accurate and easily correctable.

Image co-registration between pre- and post-ablation imaging is the basis for quantifying distances between boundaries of the tumor and ablation zone. Rigid and non-rigid co-registration techniques were used about as often and most of the co-registration methods included in this systematic review were semi-automatically. Non-rigid co-registration algorithms usually result in visually better outcomes for the entire liver, as deformational differences of the liver between the pre- and post-ablation scans are adjusted for. However, local tissue deformations as a result of thermal ablation may result in inaccurate MAM measurements. Luu et al. proposed to manually penalize local areas with large erroneous non-rigid deformations by enforcing local rigidity [50]. Similarly, Passera et al. replaced these local areas with synthetic patterns to be able to use a non-rigid co-registration approach without the undesired, erroneous deformations in the ablation zone that hamper correct MAM measurements [42]. Locally optimized co-registration between pre- and post-ablation imaging in the tumor region is the main objective. The use of local landmark placement is possible in many co-registration algorithms and may be used for this sake.

To reduce co-registration errors in a clinical setting, the pre- and post-ablation imaging are best obtained during the ablation procedure with the patient in an identical bed position and with a similar inhalation mode. Although thermal ablation could be performed using intravenous sedation, general anesthesia has the advantage of being able to use high-frequency jet ventilation or breath hold [81]. This may help reducing differences in inhalation mode, and therefore co-registration errors. It has yet to be established which scanning protocol and phase is most suitable for accurate and reproducible quantification of ablation margins.

Tissue shrinkage during ablation may be of high influence on the outcome of ablation margin quantification, with substantial tissue shrinkage rates reported in animal studies. As a result of tissue shrinkage, ablation margins may be underestimated. During the follow-up after thermal ablation, the ablation zone may shrink further on imaging [82]. Therefore, ablation margin quantification should be determined based on images acquired directly after treatment. This systematic review only included articles using CECT or MRI for immediate ablation margin evaluation. For clinical purpose, hybrid imaging with PET-CT or PET-MRI may help identifying patients at risk of developing LTP [83]. Besides direct tissue shrinkage during ablation, local edema around the ablation zone may cause the opposite effect directly surrounding the ablation necrosis, and my influence image co-registration.

The evidence available on the use of ablation margin quantification is currently based on retrospective studies with a high variability in study methodology. Both clinical factors and technical factors, in terms of image acquisition, reconstruction algorithms, and image processing play major roles in the quantification of ablation margins. A better understanding is needed of how these factors affect the outcome, and what combination of factors results in a robust and accurate method of ablation margin quantification. With this standard at hand, the correlation between measured MAM and the occurrence of LTP could ultimately be better understood and incorporated in the standard workflow.

Several prospective clinical trials are currently performed trying to bridge this gap, such as the PROMETHEUS (Netherlands Trial Register NL9713) [84], ACCLAIM (Clinicaltrials.gov: NCT03753789), COVER-ALL (Clinicaltrials.gov: NCT04083378) [85], RFA physics library – PGP (Clinicaltrials.gov: NCT04152343) and IAMCOMPLETE (Clinicaltrials.gov: NCT04123340) trials. Moreover, companies are developing solutions for ablation margin quantification and raising precision e.g. with dedicated ablation margin quantification software [31], ablation needle guidance and integrated ablation margin confirmation software [86], or an ablation system with integrated imaging co-registration and ablation margin verification software [87]. Moreover, the wider application of dual-energy CT and spectral CT may contribute to optimized tumor and ablation zone segmentation [88]. The combination of prospective clinical trials and technological advancements is what is needed to push ablation margin quantification to the next stage.

## 5 Conclusion

Ablation margin quantification is emerging to become a valuable tool in optimizing minimally invasive treatment of hepatic tumors. This systematic review shows that there is currently a high variability in ablation margin quantification methodology in terms of image co-registration, segmentation methods, and interpretation. Although the method for reaching the maximum precision in a robust way may still be unknown, the correct clinical use and interpretation will be very important as the ultimate goal is to interpret ablation margins at a millimeter level of accuracy. Optimization of scanning protocols, time reduction between pre- and post-ablation scans, and quality assessment of image co-registration are therefore of great importance.

## External funding

None.

## Sources of support

None.

## Ethical statement

This systematic review study was performed without any direct patient data. It is performed in accordance with PRISMA statement for systematic reviews. All authors contributed to the article and consent submitting to European Journal of Radiology Open for publication.

## CRediT authorship contribution statement

**de Geus-Oei Lioe-Fee:** Methodology, Supervision, Validation, Writing – review & editing. **Dijkstra Jouke:** Methodology, Supervision, Validation, Writing – review & editing. **Burgmans Mark C:** Conceptualization, Data curation, Investigation, Methodology, Project administration, Validation, Writing – review & editing. **Oosterveer Timo T M:** Data curation, Investigation, Visualization, Writing – review & editing. **Broersen Alexander:** Data curation, Methodology, Supervision, Validation, Writing – review & editing. **Hendriks Pim:** Conceptualization, Data curation, Formal analysis, Investigation, Methodology, Project administration, Validation, Writing – original draft, Writing – review & editing. **Boel Fleur:** Conceptualization, Data curation, Formal analysis, Investigation, Methodology, Validation, Visualization, Writing – original draft, Writing – review & editing.

## Declaration of Competing Interest

The authors declare that they have no known competing financial interests or personal relationships that could have appeared to influence the work reported in this paper.

## Appendix A

### Search term

("2009/01/01"[Date - Entry]: "2021/02/01"[Date - Entry]) AND.

(("Radiofrequency Ablation"[MeSH Major Topic:noexp] OR.

"Radiofrequency Ablation"[Title/Abstract] OR.

"thermal ablation"[Title/Abstract] OR.

"thermal abla*"[Title/Abstract] OR.

"Microwaves"[MeSH Major Topic] OR.

"Microwave ablation"[Title/Abstract] OR.

"Catheter Ablation"[MeSH Major Topic:noexp] OR.

"Ablation"[Title] OR.

"ablative"[Title]).

AND.

("carcinoma, hepatocellular"[MeSH Major Topic] OR.

"Liver"[MeSH Major Topic] OR.

"Liver Neoplasms"[MeSH Major Topic] OR.

"Hepatocellular carcinoma"[Title/Abstract] OR.

"hepatocellular carcinomas"[Title/Abstract] OR.

"hcc"[Title/Abstract] OR.

"Hepatic metastasis"[Title/Abstract] OR.

"liver tum*"[Title/Abstract]).

AND.

("Dehydration"[MeSH Major Topic] OR.

"Tissue shrinkage"[Title/Abstract] OR.

"shrinkage"[Title/Abstract] OR.

"shrink*"[Title/Abstract] OR.

"Contraction"[Title/Abstract] OR.

(("Algorithms"[MeSH Major Topic] OR.

"image processing, computer assisted"[MeSH Major Topic:noexp] OR.

"Image processing"[Title/Abstract] OR.

"Image Enhancement"[MeSH Major Topic] OR.

"Image Enhancement"[Title/Abstract] OR.

"Registration"[Title/Abstract] OR.

"registrat*"[Title/Abstract] OR.

"Segmentation"[Title/Abstract] OR.

"segment*"[Title/Abstract] OR.

"Computer models"[Title/Abstract] OR.

"Computer model"[Title/Abstract]).

AND ("Margins of Excision"[MeSH Major Topic] OR.

("ablat*"[Title/Abstract] AND "margin*"[Title/Abstract]) OR.

"ablation margins"[Title/Abstract] OR.

"ablation margin"[Title/Abstract] OR.

"ablative margin"[Title/Abstract] OR.

"ablated margin"[Title/Abstract] OR.

"safety margin"[Title/Abstract] OR.

"Validation"[Title/Abstract] OR.

"validat* "[Title/Abstract] OR.

("Treatment"[Title/Abstract] AND "Success"[Title/Abstract]) OR.

("Technical"[Title/Abstract] AND "Success"[Title/Abstract]) OR.

("compl*"[Title/Abstract] AND "ablat*"[Title/Abstract]) OR.

"Intraoperative Period"[MeSH Major Topic] OR.

"intra-operative"[Title/Abstract] OR.

"intraoperative"[Title/Abstract] OR.

"intra-operative"[Title/Abstract] OR.

"localization"[Title/Abstract] OR.

"localisation"[Title/Abstract]).

) NOT (("Case Reports"[Publication Type] OR "case report"[Title])).
